# Preparing for the Rollout of Pre-Exposure Prophylaxis (PrEP): A Vignette Survey to Identify Intended Sexual Behaviors among Women in Kenya and South Africa if Using PrEP

**DOI:** 10.1371/journal.pone.0129177

**Published:** 2015-06-09

**Authors:** Amy Corneli, Samuel Field, Emily Namey, Kawango Agot, Khatija Ahmed, Jacob Odhiambo, Joseph Skhosana, Greg Guest

**Affiliations:** 1 Social and Behavioral Health Sciences, FHI 360, Durham, NC, United States of America; 2 Biostatistics, FHI 360, Durham, NC, United States of America; 3 Impact Research and Development Organization, Kisumu, Kenya; 4 Setshaba Research Centre, Pretoria, South Africa; David Geffen School of Medicine at UCLA, UNITED STATES

## Abstract

**Introduction:**

Several clinical trials have demonstrated the efficacy of pre-exposure prophylaxis (PrEP) in reducing HIV risk. One concern with introducing PrEP is whether users will engage in riskier sexual behaviors.

**Methods:**

We assessed the effect that PrEP may have on sexual risk behaviors by administering a survey to 799 women in Bondo, Kenya, and Pretoria, South Africa. Participants were asked about their sexual behavior intentions twice — once as if they were taking PrEP and once as if they were not taking PrEP — within four risk situations (vignettes). They responded using a 5-point ordinal scale. We used a series of linear mixed effects models with an unstructured residual covariance matrix to estimate the between- and within-subject differences in the mean likelihood of engaging in risky sexual behavior across the PrEP and non-PrEP contexts. We also calculated the total percentage of participants who reported a greater likelihood of engaging in risky sexual behavior if taking PrEP than if not taking PrEP, by vignette.

**Results:**

We found statistically significant differences in the mean likelihood of engaging in risky sexual behavior with the between-subject comparison (-0.17, p < 0.01) and with the within-subject comparison (-0.31, p < 0.001). Depending on the vignette, 27% to 40% of participants reported a greater likelihood of engaging in risky sexual behavior if taking PrEP than if not taking PrEP.

**Conclusions:**

Our findings indicate that modest increases in risky sexual behavior could occur with PrEP. Although responses from the majority of participants suggest they would not be more likely to engage in risky sexual behavior if they took PrEP, a substantial proportion might. Programs rolling out PrEP should be prepared to assist similar women in making informed choices about reducing their risk of HIV and about their sexual health beyond HIV prevention.

## Introduction

Several clinical trials have shown that pre-exposure prophylaxis (PrEP) with oral emtricitabine/tenofovir disoproxil fumarate (FTC/TDF) is efficacious at reducing the risk of HIV acquisition [1–3]. In response, the U.S. Food and Drug Administration has approved the use of oral FTC/TDF as PrEP for HIV prevention [4]; the U.S. Centers for Disease Control and Prevention (CDC) and U.S. Public Health Service has released HIV prevention recommendations and clinical practice guidelines for using PrEP [5–6]; and the most recent guidance from the World Health Organization recommends PrEP for HIV-negative partners in HIV serodiscordant relationships and for men who have sex with men (MSM) as part of a comprehensive risk-reduction package [7].

For individuals who are unable to consistently use or negotiate the use of condoms, PrEP may be a feasible option for reducing the risk of becoming infected with HIV. A major concern with the introduction of PrEP, however, is the negative effect (i.e., increased risky sexual behavior) PrEP could have among users who are currently practicing other HIV risk-reduction measures—a concept referred to as “risk compensation” [8,9]. Previous modeling has suggested a sizeable reduction in new HIV infections with the inclusion of PrEP as an additional risk-reduction method. However, the anticipated benefit of PrEP would be diminished with the reduction of other risk-reduction behaviors, particularly if PrEP was partially effective [10,11]. PrEP has now been shown to be highly effective if taken regularly [2, 12, 13], yet concerns regarding risk compensation and HIV acquisition still remain, primarily because consistent adherence to daily PrEP may be difficult for some users and because PrEP does not completely eliminate the risk of HIV [6]. For these reasons, current guidelines recommended that providers encourage clients to use PrEP in combination with other HIV risk-reduction strategies [6]. In addition, for users who might reduce or stop the use of other HIV risk-reduction measures when taking PrEP, factors beyond HIV prevention, specifically the prevention of pregnancy and other sexually transmitted infections (STIs), must also be considered; FTC/TDF has been shown to be efficacious in reducing the risk of herpes simplex virus type 2 [14] but not any other STI.

Until recently [13,15], placebo-controlled PrEP trials have been the primary source of data on risk compensation associated with PrEP [1,2,3,16]. Increases in sexual risk behaviors were not reported among participants in the iPrEx [1,17], FEM-PrEP [16], TDF2 [3], and West Africa TDF clinical trials [18]—or among participants in the CDC’s extended safety study of TDF [19]. In the Partners PrEP Study, which was the first study to evaluate risk compensation among users of PrEP with known effectiveness, rates of unprotected sex did not increase among the HIV-negative partners and their HIV-infected study partners after the trial transitioned to an open-label study. Yet, the frequency of self-reported unprotected sex did increase between the HIV-negative partners and their other sexual partners, although there were no increases in STIs or pregnancy [15]. No risk compensation was reported among men and transgender women who have sex with men in the open-label iPrEx Ole study, another study that evaluated sexual behaviors when participants knew they were taking an efficacious PrEP product [13].

Data regarding sexual behaviors and PrEP among women in sub-Saharan Africa who may not know the serostatus of their partners are limited only to those data collected during clinical trials [3,16].

Data from placebo-controlled clinical trials on risk compensation are limited in their applicability outside of the clinical trial context because of the environment in which behaviors are carried out and data are collected [20]. Participants receive either a product of unknown effectiveness or a placebo. They are regularly counseled to continue using proven HIV prevention methods because it is unknown whether the study product will provide protection against HIV and the placebo provides no protection. Individuals’ motivation to use proven risk-reduction methods within a clinical trial might therefore differ from that outside of the clinical trial environment, where less intense counseling is provided and the effectiveness of products is known. No data are currently available on risk compensation among similar African women using a known efficacious PrEP product.

For these reasons, we conducted a mixed-methods study to prepare for the rollout of PrEP among women in communities in Kenya and South Africa. We first assessed the effect that the availability of PrEP may have on sexual risk behaviors. We then explored motivations for reducing other risk-reduction behaviors (e.g., less likely to use condoms, more likely to have sex with a new partner) among participants whose responses suggested a greater likelihood of engaging in these behaviors if they were to take PrEP. Since FTC/TDF was not approved as PrEP at the time of the study, we chose to explore sexual behavior intentions through the use of vignettes [21], an approach previously used in HIV prevention research [22–23]. The concreteness and specificity of vignettes, which are constructed to mirror real-life situations as closely as possible, provide a distinct advantage over standard hypothetical questioning. There are numerous applications of the vignette method in research [24–25], all based on presenting a constructed scenario (i.e., the vignette) to participants, who are asked to describe how they or the vignette characters would respond to that scenario. This approach allows the use of an experimental design in surveys dealing with judgments and decisions when an experimental design using the actual product is not feasible or not likely to assess implicit values, beliefs, and motivations [26–28].

Here we describe women’s sexual behavior intentions if taking PrEP. We describe elsewhere the motivations women gave for reducing the use of other risk-reduction practices if they were to take PrEP [29].

## Methods

### Vignette Survey Data Collection

**Fig 1:**
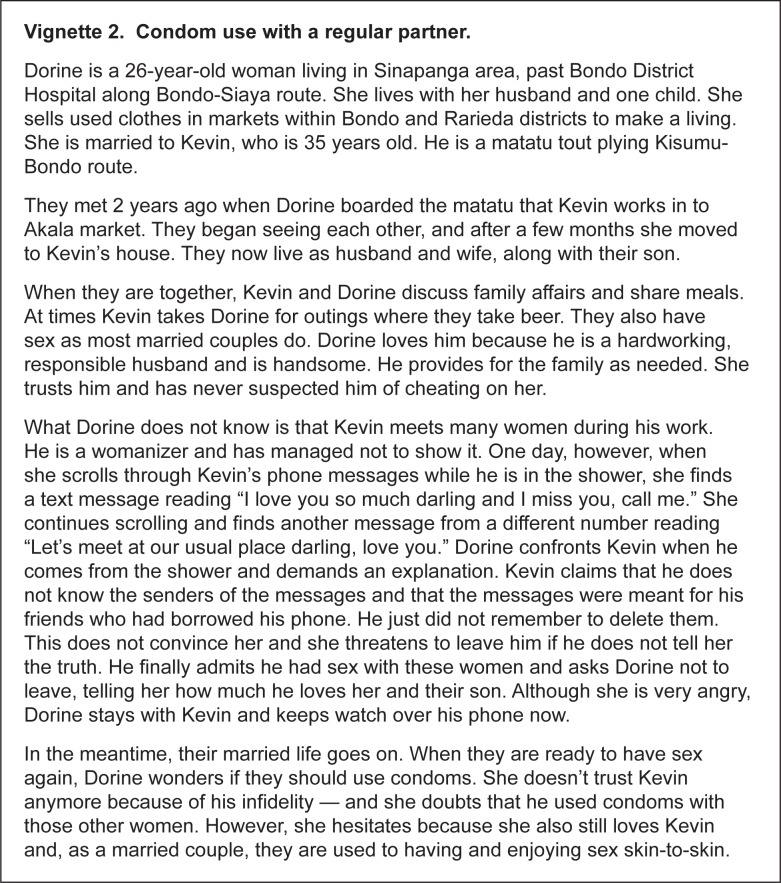
Example vignette from the Bondo site.

**Fig 2:**
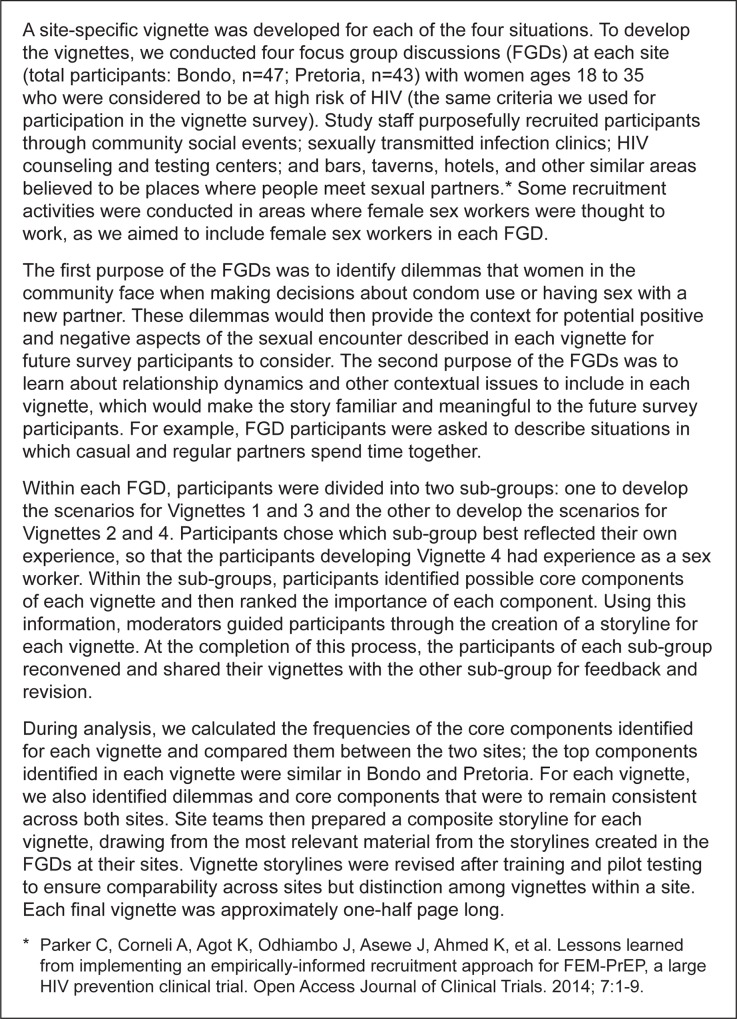
Development of the survey vignettes.

From June to November 2012, we conducted a vignette survey with women at high risk of HIV infection in Bondo, Kenya, and Pretoria, South Africa, to explore sexual behavior intentions if taking PrEP and if not taking PrEP. Bondo and Pretoria were chosen as study sites because many women in these areas were at substantial HIV risk, as demonstrated by the high incidence rates of HIV reported in FEM-PrEP (4.7 and 6.0 per 100 person-years in the placebo arm in Bondo and Pretoria, respectively) [16]. The survey was composed of four vignettes (Fig 1), each describing a situation that could place women at risk for HIV infection (Table 1). The vignettes were condom use with a casual partner (Vignette 1), condom use with a regular partner (Vignette 2), having sex with a new partner (Vignette 3), and condom use during transactional sex with a new partner (Vignette 4). Vignettes 1, 2, and 4 each introduced a dilemma in which the character in the story weighed two reasons for using a condom with the sexual partner and two reasons for not using a condom. The dilemmas were identified during focus group discussions conducted with a similar study population prior to the vignette survey (Fig 2). The same process was followed for Vignette 3, but the dilemma described the reasons for having sex and not having sex with a new partner. Although the overall sexual risk context and dilemmas for each vignette were the same at both sites, the details surrounding each vignette were tailored for the specific site.

**Table 1:** Sexual risk contexts and dilemmas in the four survey vignettes.

Vignette #		Dilemma	
	Sexual Risk Context	Reasons to not use a condom	Reasons to use a condom
1	Deciding whether to use a condom with a casual partner when having sex for the third time	She trusts partner because they have had sex two previous times.	She doesn’t know partner’s HIV status.
		He treats her well and continues to call.	She’s unsure if partner has other sexual partners.
2	Deciding whether to use a condom with a regular partner after finding out about the partner’s infidelity	She still loves her partner.	She doesn’t trust partner anymore.
		She enjoys skin-to-skin sex.	She doubts he used a condom with other sexual partners.
4	Deciding whether to use a condom during transactional sex with a new partner who is willing to pay more for sex without a condom, when the vignette character typically uses condoms with new customers	She needs the extra money.	He’s a new client whom she knows little about, and she suspects he has many partners because he comes to a place where women exchange sex for money.
		She might not get other clients today.	She worries he has HIV.
3	Deciding whether to have sex with a new partner	**Dilemma**	
		**Reasons to have sex with new partner**	**Reasons not to have sex with new partner**
		She’s been drinking and feeling tipsy and flirtatious.	She doesn’t know anything about him.
		She wants to have sex with him.	She suspects he has other partners.

During survey data collection, each vignette was read aloud to participants (because not all participants were literate). After hearing each vignette, participants were asked to respond to a single question to describe their sexual behavior intentions for that situation. Participants were asked to consider their intentions within two different contexts of PrEP use—using PrEP and not using PrEP. Participants at each site were randomized in a 1:1 ratio to the PrEP use context they would receive first, in order to control for possible effects of item sequence. After hearing Vignettes 1, 2, and 4, participants assigned first to the PrEP context were asked to respond about the likelihood of using a condom as if they were the woman in the vignette and they were taking PrEP, using a 5-point ordinal scale of “not at all likely” to “very likely.” After hearing Vignette 3, participants assigned the PrEP context were asked about the likelihood of having sex with the new partner as if they were the woman in the vignette and they were taking PrEP, also using a 5-point ordinal scale. Participants assigned first to the non-PrEP context were asked the same questions about the likelihood of using a condom or having sex with a new partner, but they were asked to respond as if they were the woman in the vignette and they were not taking PrEP. After all four vignettes were discussed for the first context of PrEP use, the interviewers read a summary of each vignette and asked participants what they would do in each vignette in the reverse context (i.e., PrEP or non-PrEP). A total of eight questions were asked in the vignette survey.

Within each PrEP use context, the order in which the four vignettes were presented in the survey was also randomized to balance participants across any possible order effect, rendering 24 different permutations of vignette orders for each context. Randomization envelopes including the vignette order were drawn in sequential order as participants were enrolled in the study. Surveys were color-coded for both context and vignette number to reduce the potential for error. At the end of the vignette survey, participants were debriefed and informed that PrEP was not currently available as an HIV risk-reduction method.

**Fig 3:**
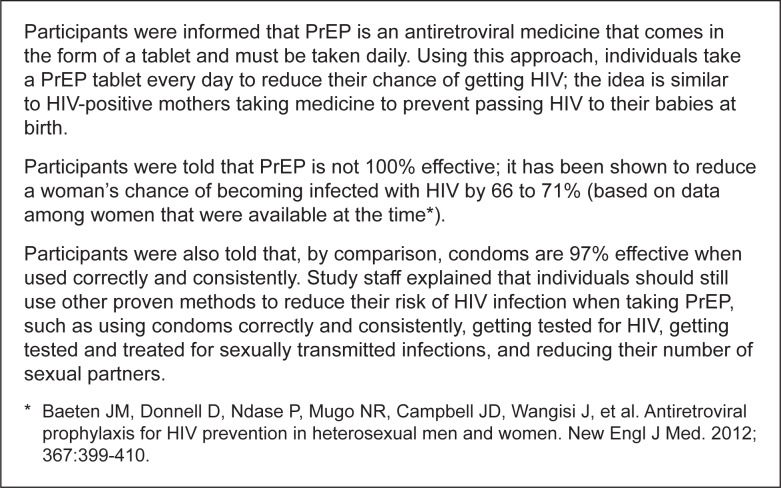
Description of the brief information on PrEP provided to participants.

Immediately prior to the vignette survey, we conducted a baseline sexual behavioral questionnaire to ask participants demographic questions as well as questions about their current sexual partnerships and sexual behaviors. We included a screening question in the sexual behavior questionnaire to determine whether a participant had exchanged sex for money within the past 12 months; Vignette 4 was presented only to those women who answered yes. We also provided the participants with brief information on PrEP, similar to the type of information that women would likely receive during risk-reduction counseling if they were given PrEP (Fig 3). This information was provided by a study staff member other than the one who administered the sexual behavior questionnaire and subsequent vignette survey.

### Eligibility and Recruitment

To be eligible to enroll in the study, women had to 1) be 18 to 35 years of age, 2) be HIV-negative, 3) have had at least one vaginal sex act in the past two weeks or more than one sexual partner in the past six months, and 4) have not received any prior counseling on PrEP. We partnered with local HIV testing and counseling centers to identify and recruit participants. During the recruitment period, center staff provided an overview of the study to all women who tested HIV-negative and referred interested women to the research staff to learn more about the study. All women who were interested received a recruitment voucher and a scheduled time to visit the study facility to participate in the survey. If participants were eligible and provided their informed consent, they were enrolled. Participants received reimbursement at the study clinic for their time and transport, based on reimbursement amounts provided in previous behavioral studies (approximately U.S. $3.50 in Bondo and U.S. $6.25 in Pretoria; participants in Bondo were also reimbursed the transportation fares they incurred).

### Ethics Statement

The research and informed consent process was reviewed and approved by the Ethics Review Committee at the Kenya Medical Research Institute (Bondo), the Pharma-Ethics Review Board (Pretoria), and the Protection of Human Subjects Committee at FHI 360 in the United States. Participants in Bondo provided verbal informed consent. We requested that verbal consent be obtained from participants in Bondo because the research was considered minimal risk and verbal consent had been previously approved for social-behavioral research in Kenya. After verbal consent was provided, study staff signed, dated, and listed the participant’s identification number on an information sheet to document that consent was obtained. For participants in Pretoria, written informed consent was required based on local ethics requirements. The same information was provided in the verbal consent information sheet used in Bondo and in the written consent form used in Pretoria. The signature line was the only difference between the two documents.

### Statistical Analysis

We hypothesized that participants in the PrEP use context would report a greater likelihood of engaging in risky sexual behavior than participants in the non-PrEP context. A sample size of 799 provided 90% power to detect a minimal difference of .23 in the mean likelihood score when using data from a single vignette and a 2-sided alpha of .05. Given that our primary exposure variable of interest (i.e., context of PrEP use) was available to use as a between- and within-subject factor, testing our hypotheses about the impact of PrEP on the likelihood of engaging in risky sexual behavior involved two qualitatively different comparisons. We therefore used a series of linear mixed effects models with an unstructured residual covariance matrix (assumed constant across study sites) to account for multiple observations per participant. The primary model included fixed effects for site, context of PrEP use, and vignette. This model was used to estimate the between- and within-subject differences in the mean likelihood of engaging in risky sexual behavior across the two contexts (i.e., PrEP and non-PrEP) and to examine the extent to which these differences were moderated by vignette, randomly assigned PrEP order, and study site. We did not include the transactional sex vignette in the analysis because it applied to only a small subset of the population. We assigned the following values to the response options for the question regarding condom use: 1 =”not at all likely,” 2 = “not likely,” 3 = “somewhat likely,” 4 = “likely,” and 5 = “very likely.” Prior to the analysis, we inverted the scores about the likelihood of having sex with a new partner (Vignette 3) so that decreasing values on the outcome measure reflected movement toward intentions for risker sexual behavior.

We also examined the degree to which the tendency to report riskier intentions in the PrEP context was moderated by the following pre-specified factors: order in which the PrEP context was presented to participants (i.e., first or second), age, marital status, education, whether a woman reported exchanging sex for money, whether a woman reported exchanging sex for non-monetary goods, having sexual partners other than a primary partner, and reported condom use with primary and other sex partners. When testing for moderation by condom use with other sex partners, the analysis was restricted to those respondents who reported having one or more other sex partners. For each potential moderating factor, we estimated a second set of models. Each of these models began with the original model specification described above, but also included fixed effects representing all possible two-, three- and four-way interactions between each potential moderator, site, vignette, and context of PrEP use. In each case, moderation was evaluated in separately estimated models. Evidence of which factors moderated the effect of the PrEP context was based on the type III tests from each model and included no adjustments for multiple testing.

Our linear mixed effects model approach also allowed us to consider the manner in which reports of risk-taking intentions from the same participants were correlated across contexts of PrEP use and vignettes. Specifically, using the primary model, we examined correlations (model-based) in risk intentions reported by the same participant in the non-PrEP context across vignettes (which we refer to as “the status quo condition”) and the difference in intentions across the two contexts of PrEP use and across vignettes (which we refer to as “risk compensation”). Lastly, as an alternative metric for evaluating the impact of PrEP, we also calculated the total percentage of participants who reported a greater likelihood of engaging in risky sexual behavior if taking PrEP than if not taking PrEP, for each vignette.

## Results

### Demographic and Behavioral Characteristics

A total of 799 HIV-negative participants were enrolled in the vignette survey; 399 participants were randomly assigned to the PrEP context first (Bondo, n = 200; Pretoria, n = 199) and 400 to the non-PrEP context first (Bondo, n = 200; Pretoria, n = 200). Demographic and sexual behavior data are described in Table 2. The mean age of participants from both Bondo and Pretoria was 24. Levels of education were different between participants in Bondo and those in Pretoria. Most participants in Pretoria (n = 378; 95%) and a substantially smaller percentage in Bondo (n = 154; 39%) had completed at least some secondary school. Among participants in Pretoria, 251 (63%) reported being unemployed or being a housewife, and 76 (19%) reporting being a student. Among participants in Bondo, the most common occupations were working as a market vendor (n = 110; 28%), in agriculture (n = 52; 13%), and in the fishing industry (n = 49; 12%); 90 participants (23%) reported being unemployed or being a housewife.

**Table 2:** Demographic characteristics, sexual partnerships, and sexual behaviors reported by vignette survey participants.

Variable	Bondo (n = 400)	Pretoria (n = 399)
*Demographic characteristics*		
Age		
Mean years	24	24
18–25 years, n(%)	270 (67.5)	276 (69.2)
26–35 years, n (%)	130 (32.5)	123 (30.8)
Education, n (%)		
Completed primary school or less	246 (61.5)	20 (5.0)
Completed some or all of secondary school	136 (34.0)	302 (75.7)
Some post-secondary certificate, diploma, degree	18 (4.5)	76 (19.0)
Adult basic education	0 (0.0)	1 (0.3)
Marital status and co-habitation, n (%)		
Not married and currently not living with partner	103 (25.8)	297 (74.4)
Not married and living with partner	6 (1.5)	87 (21.8)
Married and not living with partner	1 (.25)	1 (0.25)
Married and living with partner	263 (65.8)	13 (3.3)
Separated	11 (2.8)	0 (0.0)
Divorced	8 (2.0)	0 (0.0)
Widow	n/a	1 (.25)
Has not remarried or been inherited (Bondo only)	6 (1.5)	n/a
Has remarried or been inherited (Bondo only)	2 (.5)	n/a
Occupation, n (%)		
Market/street vendor	110 (27.5)	8 (2.0)
Fishing industry (Bondo only)	49 (12.3)	n/a
Bar, tavern, cub employee	13 (3.3)	2 (0.5)
Hotel employee	5 (1.3)	1 (0.3)
Hairdresser	23 (5.8)	16 (4.0)
Agricultural work	52 (13.0)	2 (0.5)
Office work	7 (1.8)	5 (1.3)
Student	26 (6.5)	76 (19.0)
Housewife or not employed (non-student)	90 (22.5)	251 (62.9)
Other	25 (6.3)	38 (9.5)
*Sexual partnerships and behaviors*		
Age of first sex		
≤15 years, n (%)	142 (35.5)	51 (12.8)
16–17 years, n (%)	130 (32.5)	178 (44.6)
≥18 years, n (%)	128 (32.0)	170 (42.6)
Has a primary partner, n (%)	379 (94.8)	389 (97.5)
Relationship with primary partner, n (%)	n = 378	n = 389
Husband	264 (69.8)	58 (14.9)
Boyfriend	114 (30.2)	329 (84.6)
Other	0 (0.0)	2 (0.5)
Frequency of sex with primary partner, on average per week, n (%)^1^		
≤1	50 (13.6)	57 (14.7)
2–4 times	201 (54.6)	220 (56.6)
≥5	117 (31.7)	112 (28.8)
Frequency of condom use with primary partner, in general, n (%)		
Never/rarely	212 (56.1)	178 (45.8)
Sometimes	89 (23.5)	77 (19.8)
Usually/always	77 (20.4)	134 (34.4)
Number of other male sexual partners in past month, n (%)	n = 400	n = 399
0	292 (73.0)	295 (73.9)
1	43 (10.8)	55 (13.8)
2–3	44 (11.0)	42 (10.5)
4+	21 (5.3)	7 (1.8)
Frequency of condom use with other sexual partners, in general, n (%)	n = 108	n = 104
Never/rarely	18 (16.7)	7 (6.7)
Sometimes	18 (16.7)	23 (22.1)
Usually/always	72 (66.6)	74 (71.2)
Transactional sex in the past 12 months	n = 400	n = 399
Has exchanged sex for gifts^2^	75 (18.7)	68 (17.0)
Has exchanged sex for money	74 (18.5)	51 (12.8)
Exchanges sex for money as the main source of income	57 (14.3)	36 (9.0)
Frequency of condom use with transactional sex partner for gifts, in general, n (%)	n = 70^3^	n = 66^4^
Never/rarely	14 (20.0)	15 (22.7)
Sometimes	16 (22.9)	12 (18.2)
Usually/always	40 (57.1)	39 (59.1)
Frequency of condom use with transactional sex partner for money, in general,^5^n (%)	n = 48	n = 35
Never/rarely	9 (18.8)	6 (17.1)
Sometimes	12 (25.0)	6 (17.1)
Usually/always	27 (56.2)	23 (65.7)

^1^Data missing from 10 participants in Bondo

^2^Transport, food, drink, rent, school fees, airtime

^3^Data missing from five participants

^4^Data missing from two participants

^5^Asked only to participants who reported exchanging sex for money in the past month

The majority of participants from both sites reported having a primary partner (Bondo, n = 379, 95%; Pretoria, n = 389, 98%), although the types of relationships differed between sites. In Bondo, primary partners were mostly husbands (n = 264; 70%); in Pretoria, they were mostly boyfriends (n = 329; 85%). Sex with a primary partner was frequent at both sites, with 318 (86%) participants from Bondo and 332 (85%) from Pretoria reporting having sex two or more times on average per week. In both Bondo and Pretoria, reporting to have never or rarely used a condom in the past four weeks was common with primary partners (Bondo, n = 212, 56%; Pretoria, n = 178, 46%) but not with other sexual partners (Bondo, n = 18, 17%; Pretoria, n = 7, 7%). A small percentage of women from both sites reported that they engaged in transactional sex for money (Bondo, n = 74, 19%; Pretoria, n = 51, 13%).

### Intentions for Risky Sexual Behavior

Using participants’ initial responses to the vignettes within their assigned context of PrEP use (i.e., the between-subject comparison), we found a statistically significant difference in the mean likelihood score between participants who were randomized to the PrEP context first and those who were randomized to the non-PrEP context first (-0.17, p < 0.01). In other words, participants who were “taking” PrEP had a lower mean score, suggesting a greater likelihood of engaging in risky sexual behavior. When we compared the mean difference in responses between the PrEP and non-PrEP use contexts for each participant (i.e., the within-subject comparison), the estimate of PrEP’s impact on sexual risk-taking behavior nearly doubled in magnitude and remained statistically significant (-0.31, p < 0.001); Table 3 lists the mean likelihood scores by site, PrEP sequence, PrEP context, and vignette. Depending on the vignette, 27% to 40% of participants reported a greater likelihood of engaging in risky sexual behavior if taking PrEP than if not taking PrEP. (Table 4).

**Table 3:** Mean likelihood scores by site, PrEP sequence, PrEP context, and vignette.

			Vignette					
			Casual		Regular		New	
Site	PrEP Sequence	PrEP Context	mean (sd*)	n	mean (sd)	n	mean (sd)	n
Bondo	PrEP second	non-PrEP	4.14 (1.18)	200	3.95 (1.23)	200	3.58 (1.42)	200
		PrEP	3.68 (1.37)	200	3.62 (1.34)	200	3.1 (1.41)	200
	PrEP first	non-PrEP	4.03 (1.34)	200	4.27 (1.08)	200	3.81 (1.29)	200
		PrEP	3.79 (1.39)	200	3.92 (1.26)	200	3.55 (1.4)	200
Pretoria	PrEP second	non-PrEP	3.55 (1.37)	200	3.77 (1.18)	199	3.6 (1.29)	200
		PrEP	3.31 (1.29)	200	3.47 (1.28)	200	3.14 (1.3)	200
	PrEP first	non-PrEP	3.43 (1.32)	199	3.61 (1.26)	199	3.59 (1.26)	199
		PrEP	3.36 (1.31)	199	3.63 (1.17)	199	3.31 (1.3)	199

*Abbreviation: sd = standard deviation

**Table 4:** Percentage of participants by vignette who reported a greater likelihood of engaging in risky sexual behavior in the PrEP context.^1^

Vignette	Bondo	Pretoria
	n/N (%)	n/N (%)
Casual sexual partner	120/400 (30)	121/399 (30)
Regular sexual partner^2^	135/400 (34)	108/398 (27)
New sexual partner	132/400 (33)	145/399 (36)
Sexual work partner^3^	29/73 (40)	14/50 (28)

^1^The responses among the remaining participants were either the same between the PrEP and non-PrEP contexts or showed that risk-taking behavior was lower in the PrEP context compared with the non-PrEP context. The percentage of participants who reported increased safer behaviors if taking PrEP was 13% to 22%.

^2^Data missing from one participant

^3^Data missing from two participants

Two factors were found to moderate the impact of PrEP use on risky sexual behavior. The first was the randomized order in which the PrEP context was presented to participants. The estimated impact of PrEP for those participants who were assigned to the PrEP context first was -0.2 units on the ordinal scale (p < 0.05). Conversely, for participants who were asked questions about the PrEP context second, the estimated impact was twice as large (-0.40, p < 0.001). The second factor found to moderate the impact of PrEP was type of partner; the four-way interaction between the type of partner, context of PrEP use, vignette, and site was statistically significant (p = 0.04). For participants in Bondo who reported having only a primary partner, intentions to engage in risky sexual behavior if taking PrEP were highest in Vignette 3 (i.e., the new partner vignette). However, the opposite result was observed for participants who had other current partners; intentions to engage in risky sexual behavior were the lowest in Vignette 3. We did not observe this pattern among participants in Pretoria, where the greater likelihood of engaging in risky sexual behavior were highest in Vignette 3, regardless of whether participants reported currently having a primary partner only or having a primary partner plus other partners.

The correlation in the status quo risk-taking reported by the same participant across Vignettes 1 and 2 (i.e., the casual and regular partner vignettes) and without PrEP use was *ρ* = .34 (Table 5). In contrast, the reported status quo risk-taking for Vignette 3 (i.e., the new partner vignette) was only weakly correlated with regular partners (*ρ* = .09) and casual partners (*ρ* = .01). In the case of risk compensation, a similar though less pronounced pattern of results was obtained. Participants whose responses suggested they will engage in riskier sexual behaviors with casual partners if taking PrEP were slightly more likely to also engage in risker sexual behaviors with their regular partners (*ρ* = .26) when taking PrEP. However, the pair of correlations involving the new partner vignette were both close to zero for regular partners (*ρ* = .02) and casual partners (*ρ* = .03).

**Table 5:** Within-subject correlations from linear mixed effects model.

	Response under non-PrEP context (status quo condition)			Difference between PrEP use contexts (risk compensation)		
	*Vignette*			*Vignette*		
*Vignette*	Casual	Regular	New	Casual	Regular	New
**Casual**	1			1		
**Regular**	0.34	1		0.26	1	
**New**	0.01	0.09	1	0.03	0.02	1

## Discussion

Our overall survey findings indicate that increases in risky sexual behavior could occur with PrEP rollout. However, the differences we observed in the likelihood of engaging in risky sexual behavior across the two PrEP contexts were quite modest; the largest estimated effect was less than a half-point decrease on a 5-point ordinal scale. Although the responses given by the majority of participants for each vignette suggest that they would not engage in risky sexual behavior if they were to take PrEP—which is similar to the findings on risk compensation from placebo-controlled clinical trials and open-label studies on PrEP [1, 3, 13, 15–19]—a substantial proportion might. These findings highlight the need to prepare for the possibility that some women might change their sexual behaviors when using PrEP. For women who adhere daily to PrEP, their risk of HIV acquisition will be significantly reduced. Thus, the behaviors described here may not be as risky with respect to HIV prevention (although U.S. guidelines promote the use of PrEP and condoms for the most protection against HIV [6]). However, consideration must be given to the prevention of several other STIs and pregnancy, particularly if condoms are discontinued in lieu of PrEP. Moreover, the importance of daily adherence and persistence with PrEP must be emphasized.

We found little evidence overall that increases in risky sexual behavior would be more likely to happen in certain risk situations or based on participants’ demographic information or reported sexual behaviors. The low within-subject correlations we observed suggest that although women might have a general intention to increase risky sexual behavior if taking PrEP, different women may increase their risk-taking behavior in one risk situation but not in another. For example, the subset of participants who reported a greater likelihood of engaging in risky sexual behavior in response to taking PrEP in one vignette (e.g., not using a condom with a regular partner) were often not the same women who would do so in another vignette (e.g., not using a condom with a casual partner) and vice versa. Similarly, knowing a participant’s reported likelihood of using condoms with regular and casual partners if taking PrEP says very little about their intentions for having sex with a new partner if taking PrEP. Not surprisingly, these findings suggest that women perceive their risk situations differently and may act according to their own examination of risks and benefits. The findings from Bondo also suggest that for some women who have only a primary partner, PrEP may provide a sense of protection against HIV that could increase their comfort with engaging in sex with new partners.

Our study has several limitations. First, although the vignette survey approach provides an advantage over standard hypothetical questioning, self-reports of hypothetical rather than actual behavior (i.e., taking PrEP) still have their limitations. For example, it is possible that not all of the women in the study personally related to all the vignettes presented in the survey. Second, participants may have given socially desirable responses and over-reported their intentions to use condoms in either context of PrEP use, particularly given the information that was provided about PrEP at the beginning of the study and the recommendation that individuals continue to use other HIV risk-reduction measures, such as condoms, when using PrEP. Although this information may have created socially desirable responses among some, such information was necessary as it mimics information that would likely be presented to women during PrEP counseling in the future. Conversely, because this study was not associated with a PrEP trial or demonstration project, participants may have been less inclined to report that their current HIV risk-reduction practices would not change if they were taking PrEP. Third, in the within-subject analysis, the detection of an effect of the order in which participants were assigned to the PrEP or non-PrEP context introduces some uncertainty about the degree to which the availability of PrEP may affect sexual risk-taking behaviors. The pronounced order effect may have occurred because participants’ initial responses were generally high on the ordinal scale (i.e., the mean was close to 4 on a 5-point scale); the high responses could have been because of a general inclination to provide socially desirable responses (i.e., engage in less risky behaviors). For participants who were assigned the PrEP context first, their subsequent answers to the non-PrEP context were typically upward on the ordinal scale (i.e., toward safer sex behavior); for participants receiving the non-PrEP context first, their subsequent answers to the PrEP context were typically lower on the ordinal scale (i.e., toward more risky sexual behavior). Because the mean response in the first context was closer to the ceiling of the ordinal scale, participants who were adjusting downward had further to go and therefore tended to report the largest changes in risk-taking behavior. However, regardless of the comparison used (i.e., between- or within-subject), the impact of PrEP on the use of risk-reduction practices was consistent in its direction and was statistically significant in both analyses. Lastly, a small percentage of women reported that they exchanged sex for money; hence, we were not able to include their responses to Vignette 4 in the overall model due to the small sample size.

Risk compensation can be truly assessed only when PrEP is provided and used as part of a woman’s comprehensive HIV risk-reduction strategy. Multiple demonstration projects are now being planned or are under way globally [30] to assess whether individuals are interested in using and adhering to daily PrEP and whether current HIV risk-reduction behaviors will change as a result of initiating and taking PrEP. These studies are likely adequately powered to detect small changes in sexual risk-taking behavior and assess the overall impact of these changes on women’s overall sexual health.

In conclusion, HIV prevention programs that will eventually roll out PrEP to women should be prepared to address the potential that some women who use PrEP will reduce or not use other HIV risk-reduction measures. Findings from follow-up qualitative interviews with survey participants whose responses suggested they had a greater likelihood of engaging in risky sexual behavior if they start taking PrEP describe the reasons that they and other women in their communities may stop their current HIV risk-reduction practices if using PrEP [29]. Together these data can inform future risk-reduction guidance for women using PrEP, to assist them in making informed choices about reducing their risk of HIV acquisition and about their overall sexual health beyond HIV prevention [31].

## References

[1] Grant RM, Lama JR, Anderson PL, McMahan V, Liu AY, Vargas L, et al Preexposure chemoprophylaxis for HIV prevention in men who have sex with men. New Engl J Med. 2010; 363:2587–99. 10.1056/NEJMoa1011205 21091279 PMC3079639

[2] Baeten JM, Donnell D, Ndase P, Mugo NR, Campbell JD, Wangisi J, et al Antiretroviral prophylaxis for HIV prevention in heterosexual men and women. New Engl J Med. 2012; 367:399–410. 10.1056/NEJMoa1108524 22784037 PMC3770474

[3] Thigpen MC, Kebaabetswe PM, Paxton LA, Smith DK, Rose CE, Segolodi TM, et al Antiretroviral preexposure prophylaxis for heterosexual HIV transmission in Botswana. New Engl J Med. 2012; 367:423–34. 10.1056/NEJMoa1110711 22784038

[4] U.S. Food and Drug Administration (FDA). FDA Approves First Drug for Reducing the Risk of Sexually Acquired HIV Infection. Silver Spring, MD: U.S. Food and Drug Administration 2012 Available: http://www.fda.gov/NewsEvents/Newsroom/PressAnnouncements/ucm312210.htm.

[5] Centers for Disease Control and Prevention, Health Resources and Services Administration, National Institutes of Health, American Academy of HIV Medicine, Association of Nurses in AIDS Care, International Association of Providers of AIDS Care, the National Minority AIDS Council, and Urban Coalition for HIV/AIDS Prevention Services. *Recommendations for HIV Prevention with Adults and Adolescents with HIV in the United States*, *2014* Available: http://stacks.cdc.gov/view/cdc/26062. Accessed 11 December 2014.

[6] U.S. Public Health Service. Preexposure Prophylaxis for the Prevention of HIV Infection in the United States– 2014: A Clinical Practice Guideline. U.S. Department of Health and Human Services and U.S. Centers for Disease Control and Prevention. 2014. Available: http://www.cdc.gov/hiv/pdf/PrEPguidelines2014.pdf

[7] World Health Organization. Consolidated Guidelines on HIV Prevention, Diagnosis, Treatment and Care for Key Populations. Geneva, Switzerland: World Health Organization 2014 Available: http://www.who.int/hiv/pub/guidelines/keypopulations/en/ 25996019

[8] Eaton LA, Kalichman S. Risk compensation in HIV prevention: implications for vaccines, microbicides, and other biomedical HIV prevention technologies. Curr HIV/AIDS Rep. 2007; 4:165–72. 18366947 10.1007/s11904-007-0024-7 PMC2937204

[9] Cassell MM, Halperin DT, Shelton JD, Stanton D. Risk compensation: the Achilles' heel of innovations in HIV prevention? BMJ. 2006; 332:605–7. 16528088 10.1136/bmj.332.7541.605 PMC1397752

[10] Vissers D, Voeten HA, Nagelkerke NJ, Habbema JD, de Vlas SJ. The impact of pre-exposure prophylaxis (PrEP) on HIV epidemics in Africa and India: A simulation study. PLOS One. 2008; 3:e2077 10.1371/journal.pone.0002077 18461185 PMC2367053

[11] Abbas UL, Anderson RM, Mellors JW. Potential impact of antiretroviral chemoprophylaxis on HIV-1 transmission in resource-limited settings. PLOS One. 2007; 2:e875 17878928 10.1371/journal.pone.0000875 PMC1975470

[12] Donnell D, Baeten JM, Bumpus NN, Brantley J, Bangsberg DR, Haberer JE, et al HIV protective efficacy and correlates of tenofovir blood concentrations in a clinical trial of PrEP for HIV prevention. J Acquir Immune Defic Syndr. 2014; 66:340–8. 10.1097/QAI.0000000000000172 24784763 PMC4059553

[13] Grant RM, Anderson PL, McMahan V, Liu A, Amico KR, Mehrotra M, et al Uptake of pre-exposure prophylaxis, sexual practices, and HIV incidence in men and transgender women who have sex with men: a cohort study. Lancet infect Dis. 2014; 14:820–29. 10.1016/S1473-3099(14)70847-3 25065857 PMC6107918

[14] Celum C, Morrow RA, Donnell D, et al Daily oral tenofovir and emtricitabine-tenofovir preexposure prophylaxis reduces herpes simplex virus type 2 acquisition among heterosexual HIV-1-uninfected men and women: a subgroup analysis of a randomized trial. Ann Intern Med. 2014 7 1;161(1):11–9. 10.7326/M13-2471 24979446

[15] Mugwanya KK, Donnell D, Celum C, Thomas KK, Ndase P, Mugo N, et al Sexual behaviour of heterosexual men and women receiving antiretroviral pre-exposure prophylaxis for HIV prevention: a longitudinal analysis. Lancet Infect Dis. 2013; 13:1021–8. 10.1016/S1473-3099(13)70226-3 24139639 PMC3920826

[16] Van Damme L, Corneli A, Ahmed K, Agot K, Lombaard J, Kapiga S, et al Preexposure prophylaxis for HIV infection among African women. N Engl J Med. 2012; 367:411–22. 10.1056/NEJMoa1202614 22784040 PMC3687217

[17] Marcus JL, Glidden DV, Mayer KH, Liu AY, Buchbinder SP, Amico KR, et al No evidence of sexual risk compensation in the iPrEx trial of daily oral HIV preexposure prophylaxis. PLOS One. 2013; 8:e81997 10.1371/journal.pone.0081997 24367497 PMC3867330

[18] Guest G, Shattuck D, Johnson L, Akumatey B, Clarke EE, Chen PL, et al Changes in sexual risk behavior among participants in a PrEP HIV prevention trial. Sex Transm Dis. 2008; 35:1002–8. 19051397

[19] Liu AY, Vittinghoff E, Chilag K, Mayer K, Thompson M, Grohskopf L, et al Sexual risk behavior among HIV-uninfected men who have sex with men participating in a tenofovir preexposure prophylaxis randomized trial in the United States. J Acquir Immune Defic Syndr. 2013; 64:87–94. 10.1097/QAI.0b013e31828f097a 23481668 PMC3904758

[20] Kim SC, Becker S, Dieffenbach C, Hanewall BS, Hankins C, Lo YR, et al Planning for pre-exposure prophylaxis to prevent HIV transmission: challenges and opportunities. J Int AIDS Soc. 2010; 13:24 10.1186/1758-2652-13-24 20624303 PMC2914050

[21] Fleming P, Stalker M. In focus: using vignettes in public health research In: Guest G, Namey EE, eds. Public Health Research Methods. Los Angeles, CA: Sage Publications, Inc; 2014 pp. 611–614.

[22] Hughes R. Considering the vignette technique and its application to a study of drug injecting and HIV risk and safer behaviour. Sociology of Health & Illness. 1998; 20:381–400.

[23] Hennessy M, MacQueen K, McKirnan DJ, Buchbinder S, Judson F, Douglas JM, et al A factorial survey study to assess the acceptability of HIV vaccine trial designs. Control Clin Trials. 1996; 17:209–20. 8877256 10.1016/0197-2456(95)00155-7

[24] Rossi P, Nock S. Measuring Social Judgments: The Factorial Survey Approach. Beverly Hills, CA: Sage Publications 1982.

[25] Finch J. The vignette technique in survey research. Sociology. 1987; 21:105 3574160

[26] Hox J, Kreft I, Hermkens P. The analysis of factorial surveys. Sociological Methods & Research. 1991; 19:493.

[27] Ludwick R, Zeller RA. The factorial survey: an experimental method to replicate real world problems. Nurs Res. 2001; 50: 129–33 11302293 10.1097/00006199-200103000-00009

[28] Taylor B. Factorial surveys: using vignettes to study professional judgment. British Journal of Social Work. 2006; 36:1187.

[29] Corneli A, Namey E, Ahmed K, Agot K, Skhosana, Odhiambo J, et al. Motivations for reducing other HIV risk-reduction practices if taking pre-exposure prophylaxis: findings from a qualitative study among women in Kenya and South Africa. AIDS Patient Care STDs. In press. 10.1089/apc.2015.0038 PMC4553377 26196411

[30] AVAC. Ongoing and Planned PrEP Trials and Demonstration Projects, as of August 2013. Available: http://www.avac.org/ht/a/GetDocumentAction/i/3113.

[31] Corneli A, Yacobson I, Namey E, Agot A, Ahmed K, Skhosana J, et al. Guidelines on Informed-Choice Counseling for Women Using Pre-Exposure Prophylaxis (PrEP). The HIV Research for Prevention Conference, Cape Town, South Africa, October, 28–31, 2014. Abstract P46.02.

